# Crypt residing bacteria and proximal colonic carcinogenesis in a mouse model of Lynch syndrome

**DOI:** 10.1002/ijc.33028

**Published:** 2020-05-18

**Authors:** Michaela Lang, Maximilian Baumgartner, Aleksandra Rożalska, Adrian Frick, Alessandra Riva, Michael Jarek, David Berry, Christoph Gasche

**Affiliations:** ^1^ Division of Gastroenterology and Hepatology, Department of Internal Medicine 3 Medical University of Vienna Vienna Austria; ^2^ Centre for Microbiology and Environmental Systems Science, Department of Microbiology and Ecosystem Science, Division of Microbial Ecology University of Vienna Vienna Austria; ^3^ Genome Analytics, Helmholtz Centre for Infection Research Braunschweig Germany; ^4^ The Joint Microbiome Facility of the Medical University of Vienna and the University of Vienna Vienna Austria

**Keywords:** colorectal cancer, inflammatory bowel disease, Lynch syndrome, microbiota

## Abstract

Colorectal cancer is a multifactorial disease involving inherited DNA mutations, environmental factors, gut inflammation and intestinal microbiota. Certain germline mutations within the DNA mismatch repair system are associated with Lynch syndrome tumors including right‐sided colorectal cancer with mucinous phenotype and presence of an inflammatory infiltrate. Such tumors are more often associated with bacterial biofilms, which may contribute to disease onset and progression. Inflammatory bowel diseases are also associated with colorectal cancer and intestinal dysbiosis. Herein we addressed the question, whether inflammation can aggravate colorectal cancer development under mismatch repair deficiency. MSH2^loxP/loxP Vill‐cre^ mice were crossed into the IL‐10^−/−^ background to study the importance of inflammation and mucosal bacteria as a driver of tumorigenesis in a Lynch syndrome mouse model. An increase in large bowel tumorigenesis was found in double knockout mice both under conventional housing and under specific pathogen‐free conditions. This increase was mostly due to the development of proximal tumors, a hotspot for tumorigenesis in Lynch syndrome, and was associated with a higher degree of inflammation. Additionally, bacterial invasion into the mucus of tumor crypts was observed in the proximal tumors. Inflammation shifted fecal and mucosal microbiota composition and was associated with enrichment in *Escherichia‐Shigella* as well as *Akkermansia*, *Bacteroides* and *Parabacteroides* genera in fecal samples. Tumor‐bearing double knockout mice showed a similar enrichment for *Escherichia‐Shigella* and *Parabacteroides*. *Lactobacilli*, *Lachnospiraceae* and *Muribaculaceae* family members were depleted upon inflammation. In summary, chronic inflammation aggravates colonic tumorigenesis under mismatch repair deficiency and is associated with a shift in microbiota composition.

## Abstract 2

**What's new?**

DNA mismatch repair deficiency and longstanding inflammatory bowel disease are predisposing factors for colorectal cancer. Greater knowledge of these factors is relevant particularly for understanding carcinogenesis in the proximal colon, a cancer hotspot in patients with Lynch syndrome. Here, using a novel double knockout mouse model (MSH2^loxP/loxP Vil‐cre^ x IL‐10) to investigate the combined role of inflammation and mucosal bacteria in Lynch syndrome, the authors show that proximal colonic tumorigenesis in mismatch repair‐deficient mice is aggravated under chronic inflammation. Proximal colon tumorigenesis was accompanied by bacterial invasion into tumor crypts. The findings shed light on colonic tumorigenesis mechanisms in MSH2^loxP/loxP Vil‐cre^ x IL‐10 mice.

**Abbreviations**
5‐ASA: 5‐aminosalicylic acid
AOM: azoxymethane
CRC: colorectal cancer
DKO: double knockout
DSS: dextran sulphate sodium
FAP: familial adenomatous polyposis
FISH: fluorescence in situ hybridization
FoV: fields of view
H&E: hematoxylin and eosin
IL‐10: interleukin 10
LB: large bowel
MSI: microsatellite instability
MSH2: MutS homolog 2
OTU: operational taxonomic unit
PCR: polymerase chain reaction
SB: small bowel
SPF: specific pathogen‐free

## 1 INTRODUCTION

Genetic disorders such as Lynch syndrome and familial adenomatous polyposis (FAP) lead to an earlier development of intestinal cancer compared to sporadic colorectal cancer (CRC). Also, the lifetime risk to develop CRC is much higher. In Lynch syndrome, the most prevalent inherited form of CRC, synchronous and metachronous tumors are often found. The tumors, typically located in the right‐sided colon, are often mucinous and characterized by an inflammatory infiltrate.^1^, ^2^ Also, inflammatory bowel disease patients have a higher incidence of synchronous and metachronous colorectal tumors in the proximal colon.^3^ Herein we investigated whether intestinal inflammation can aggravate genetically driven colorectal carcinogenesis with respect to anatomical tumor localization and changes in intestinal microbiota.

Right colonic tumor localization is recognized in specific subtypes of CRC featuring different mutational etiologies of colorectal carcinogenesis.^1^, ^3^ The colon is also home to the highest concentration of microbes, which are implicated in CRC development.^4^, ^5^, ^6^, ^7^, ^8^, ^9^ Mucus integrity, the epithelial barrier, bowel inflammation, the environment and host genetic factors are also thought to contribute to disease pathogenesis.

It is well know that IL‐10^−/−^ mice tend to develop colitis and tumors in the proximal colon^10^, ^11^, ^12^ whereas mice treated with the carcinogen azoxymethane (AOM) and the chemical colitogen dextran sulfate sodium (DSS) develop more distal colonic tumors.^13^ Similarly, MSH2^loxP/loxP Vil‐cre^ mice, which display intestinal epithelial mismatch repair deficiency, show a higher percentage of proximal colonic tumors, which are often located close to the ileocecal valve. These mice have been established as a mouse model for Lynch syndrome.^14^, ^15^

In humans, bacterial biofilms and mucus invasive bacteria have recently been reported to be a characteristic feature of right‐sided tumors.^5^, ^16^, ^17^ Biopsies of the normal‐appearing mucosa from these patients also display biofilms, which are uncommonly seen in patients with distal tumors or healthy controls. In FAP, biofilms are not restricted to polyps, but are present as patchy biofilms within the normal colonic mucosa.^6^ These biofilms predominately contain tumorigenic bacteria such as *pks + Escherichia coli* and enterotoxigenic *Bacteroides fragilis*, expressing colibactin and *Bacteroides fragilis* toxin, respectively. The authors suggest that biofilm formation may be an early event in the progression of hereditary CRC. A recent study even demonstrated that bacterial inocula from biofilms of healthy subjects induce tumor formation in APC^Min^ mice.^4^

The importance of biofilms, mucosal bacteria and/or a direct contact and adherence of bacteria to colonic epithelial cells is not only recognized for subtypes of CRC, but is also a prominent feature of inflammatory bowel diseases.^18^, ^19^ In DSS‐treated or IL‐10^−/−^ mice, the number of crypts invaded by bacteria increase upon inflammation despite concurrent thickening of the mucus layer.^18^ In general, a higher number of crypts with bacteria is found in the proximal colon compared to the distal colon, which, in a healthy gut, might be also of physiological relevance, for example, for fermentation processes.^18^, ^20^ Whether this closer interaction of certain bacteria with the intestinal epithelium in the proximal colon might also foster inflammation and (colitis‐associated) tumor development, remains in debate. For our study, we cross‐bred MSH2^loxP/loxP Vil‐cre^ mice into the IL‐10^−/−^ background to study the relevance of inflammation, intestinal tumor localization and bacterial proximity as important cofactors for tumor development under mismatch repair deficiency.

## 2 MATERIALS AND METHODS

### 2.1 Mice

MSH2^loxP/loxP Vil‐cre^ x IL‐10^−/−^ double knockout mice (DKO) mice on a C57BL/6J background were developed by crossing IL‐10^−/−^ and MSH2^loxP/loxP Vil‐cre^ single KO mice. Single KO mice were kept as controls. Genotype was confirmed by tail DNA genotyping using PCR for MSH2 and IL‐10 and real‐time PCR for Cre and the housekeeping gene 36b4 (Table S1). Mice were either kept under conventional housing at the Institute of Biomedical Research (Medical University of Vienna, Austria) or under specific pathogen‐free (SPF) conditions at the Division of Laboratory Animal Science and Genetics (Himberg, Austria) under 12 hours light/dark cycles, and chow and water was available ad libitum. At experimental start mice were 7‐9 weeks old (n = 9‐19 mice/group). Weight was recorded for conventionally housed mice at least once a week. Conventionally housed DKO mice were randomly divided into three groups and fed either a standard chow (1534‐00, SM R/M, Ssniff, Soest, Germany), a chow containing 500 mg/kg 5‐aminosalicylic acid (5‐ASA) or were gavaged three times a week with 30 mg tofacitinib/kg body weight (corresponding to 48.45 mg tofacitinib citrate, CP‐690550, Selleckchem) in 0.5% methylcellulose, 0.025% Tween 20 in dH_2_O (solvent) for 25 weeks. Conventionally housed DKO mice that did not receive tofacitinib were gavaged with the solvent only. IL‐10^−/−^ and MSH2^loxP/loxP Vill‐cre^ single KO mice served as controls (n = 9‐12 mice per group) and were also gavaged with the solvent. At 32‐34 weeks, mice were euthanized. Animal experiments were performed once, as our ethical committee does not allow a plain repetition of the experiments. All experiments were executed in accordance with the Austrian and European law, defined by the Good Scientific Practice guidelines of the Medical University of Vienna (BMWF‐66.009/0324‐WF/V/3b/2016).

### 2.2 Histology and immunohistochemical analyses

Upon euthanasia intestines were removed, flushed with cold PBS and the last flush of the large bowel was collected as mucus‐enriched fraction. Intestines, livers and spleens were fixed in 10% formalin for 24 hours with subsequent paraffin embedding. From a subset of mice, the large bowel was processed with fecal matter inside by tying the ends of the colon with a thread. These intestines were fixed in methacarn solution (60% methanol, 30% chloroform and 10% glacial acetic acid) over night at 4°C, washed three times in 70% ethanol and processed for paraffin embedding. Samples were stored at 4°C. Then, 3‐6 μm thick intestinal tissue sections were hematoxylin and eosin (H&E) stained and analyzed for intestinal tumor number, localization (rectum, distal or proximal large bowel, ileocecal valve, cecum or small bowel), cancer stage (adenoma/dysplasia, adenocarcinoma—here defined by a penetration of the muscularis mucosae) and the presence of bacteria within the mucus of tumor crypts. Gram staining of intestines was performed as described in the manufacturer's protocol (77730, Gram Staining Kit, Sigma‐Aldrich).

Large intestinal inflammation was scored throughout the colon by evaluating immune cell infiltration and by counting the number of crypt abscesses in seven fields of view (FoV) using a ×10 objective (score 0 = no inflammation, 1 = mild and patchy inflammatory infiltrate, 2 = moderate inflammatory infiltrate, low number of crypt abscesses: <average 2.5/×10 FoV), 3 = severe inflammation, high number of crypt abscesses: >2.5/×10 FoV). Lobular and portal inflammation was scored by counting the number of foci with >5 inflammatory cells in 15 FoV using a ×20 objective. Microscopic analysis was performed on an Olympus BX51 microscope. Images were taken with the CellsSens Dimension software (Olympus, Shinjuku, Tokio, Japan).

Immunohistochemical staining of liver macrophages was performed using F4/80 and biotinylated goat antirat secondary antibody (Table S2) and standard staining procedures. Briefly, slides were deparaffinized in xylol, rehydrated in a decreasing alcohol series, antigens were retrieved in 10 mM citrate buffer, pH 6 and slides were blocked with 10% goat serum, 3% BSA in TRIS buffer. F4/80 antibody incubation was performed overnight at 4°C, followed by biotinylated secondary antibody, avidin‐biotin‐HRP complex, 3,3′‐diaminobenzidine (DAB), hematoxylin staining and embedding in Histofluid. The number of liver F4/80^+^ cells in 10 FoV (n_DKO_ = 8 mice, n_IL‐10_ = 8, n_MSH2_ = 11) was counted using a ×40 objective.

### 2.3 IL‐18 immunoassay

Blood from conventionally housed mice was collected via cardiac puncture under terminal anesthesia and serum was stored at −80°C. IL‐18 levels in serum were determined using the ProcartaPlex Multiplex Immunoassay (Affymetrix) on a Bioplex 200 System (Bio‐Rad Laboratories) following manufacturer's instructions.

### 2.4 Fluorescence in situ hybridization and immunofluorescence staining

10 μm thick intestinal tissues sections were deparaffinized in xylol and rehydrated using a descending alcohol series. Then, 500 nM EUB338‐I‐III‐6‐FAM, a 16S rRNA‐targeted DNA oligonucleotide probe mix (Biomers, Ulm, Germany) to detect all bacteria by FISH (for sequences see Table S3), were applied in hybridization buffer (900 mM NaCl, 20 mM TRIS‐HCl, pH 7.2, 0.01% SDS) at 46°C for 2 hours and washed in wash buffer (900 mM NaCl, 20 mM TRIS‐HCl, pH 7.2) at 48°C for 10 minutes. For immunofluorescence staining, MUC2 antibody was applied overnight followed by incubation with AlexaFluor 594 secondary antibody (Table S2) in wash buffer for 1 hour. To visualize DNA, Hoechst 33258 staining was performed. Slides were embedded in CitiFluor AF1 (Science Services, Munich, Germany). Images were acquired on an inverted DMI 6000 confocal laser scanning microscope (Leica) equipped with a 405 nm UV diode and a Leica supercontinuum white light laser using a ×60 glycerol objective and the software Leica Application Suite AF.

### 2.5 DNA isolation

Fecal pellets and mucus‐enriched fractions were sampled at experimental endpoint from mice kept under conventional housing. Samples were shock‐frozen in liquid nitrogen and stored at −80°C. DNA was extracted from fecal pellets and mucus‐enriched fractions using a phenol‐chloroform protocol with bead beating, as performed previously.^21^ DNA was precipitated with 0.1 volumes 3 M Na‐acetate and 0.6 volumes of ice‐cold isopropanol, washed in ethanol, dried and dissolved in nuclease‐free H_2_O.

### 2.6 16S rRNA gene amplicon sequencing of intestinal microbiota

15 ng of DNA from fecal or mucus‐enriched samples were subjected to a two‐step PCR amplification targeting the V3‐V4 region of the 16S rRNA gene of most bacteria (primers 341F and 785R, Table S1).^21^, ^22^ This protocol minimizes barcoded primer‐associated bias and produces data that can reliably reproduce phylotype abundances.^23^ Both PCRs were carried out in triplicates. PCR reactions were pooled after the first 20 cycles, 10 cycles were applied for the second PCR, yielding 464 bp amplicons with an eight nucleotide sample‐specific barcode sequence added. PCR products were pooled, purified with Agencourt AMPure beads (Beckman Coulter Genomics, Danvers, MA) and quantified with PicoGreen (Quant‐iT PicoGreen, Invitrogen, Carlsbad, CA). Equimolar concentrations of barcoded amplicons were pooled for library preparation. Library for sequencing of fecal and mucus‐enriched samples was generated using NEBNext Ultra DNA Library Prep Kit for Illumina (NEBNext, E7370S) following the manufacturer's instruction without size selection. Then, 10 nM of the library with 20% phiX was used for sequencing. Sequencing was performed on the MiSeq Personal Sequencer (Illumina Inc., San Diego, CA) using MiSeq Reagent Kits v3 (Illumina). Sequencing was done to 300 cycles in both directions.

Sequence data were sorted into libraries using the eight nucleotide sample‐specific barcode and primer using a custom‐made in‐house script, quality‐filtered according to the Earth Microbiome Project guidelines and paired end reads were concatenated.^24^ Reads were then clustered into species‐level operational taxonomic units (OTUs) of 97% sequence identity, checked for chimeras using USEARCH and were taxonomically classified using the Ribosomal Database Project naive Bayesian classifier.^25^, ^26^

### 2.7 Statistics

Statistical analysis was performed using SPSS software version 23.0 (IBM). Tumor number, number of tumor crypts colonized by bacteria, the number of F4/80+ liver macrophages and IL‐18 serum levels were analyzed using Independent Samples Kruskal‐Wallis test to compare all groups with each other and Independent‐Samples Mann‐Whitney *U*‐test to compare two groups with each other. Tumor and inflammation incidence between groups was analyzed using Pearson's χ^2^‐test. Pearson's correlation was used to correlate the number of large bowel tumors with intestinal inflammation. Statistics for microbiome analysis was done using modified Rhea scripts.^27^ To analyze similarity of microbial profiles generalized UniFrac distances were used.^28^ Significant separation of groups was assessed with permutational multivariate analysis of variance (perMANOVA) using adonis from the vegan package.^29^ To compare bacterial abundances Kruskal‐Wallis for overall and Wilcoxon Rank Sum test for pairwise comparison was used. Data on bacterial genera abundance was centered and log‐ratio transformed for Pearson's correlation, FDR was used for multiple comparison corrections. Values of *P* were considered statistically significant if less or equal to .05 (*P ≤ .05; **P < .01; ***P < .001).

## 3 RESULTS

### 3.1 IL‐10 deficiency drives colonic tumorigenesis in MSH2^loxP^ ^/loxP Vil‐cre^ mice

**FIGURE 1:**
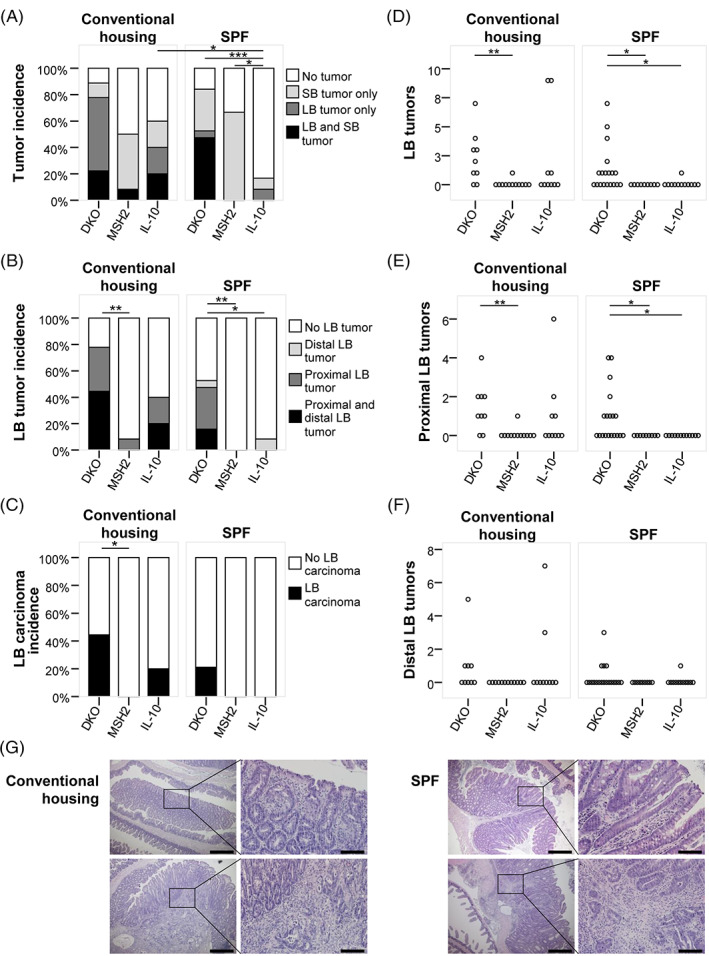
Tumor incidence and large bowel tumor multiplicity. The number of tumors (adenomas and carcinomas) in the small bowel (SB) and large bowel (LB) of DKO (n_conventional housing_ = 9; n_SPF_ = 19), MSH2^loxPloxP Vil‐cre^ (n_conventional housing_ = 12; n_SPF_ = 9) and IL‐10^−/−^ (n_conventional housing_ = 10; n_SPF_ = 12) mice was evaluated at 32‐34 weeks of age. A, Tumor incidence, accounting for the number of mice with SB and/or LB tumors, is shown. B, LB tumor incidence, accounting for proximally and/or distally located LB tumors. C, Carcinoma incidence in the LB. Tumor multiplicity (D) in the LB, (E) in the proximal colon and (F) in the distal colon is depicted. G, Representative images of adenomas (top panels) and carcinomas (bottom panels) from H&E stained histological sections of mice from different housing conditions (Scale bars: low magnification images 500 μm; high magnification images: 100 μm). Statistical analysis: Pearson χ^2^‐test for incidence; Independent samples Mann‐Whitney *U* test to compare tumor multiplicity between two groups; *P < .05, **P < .01, ***P < .001

To study the impact of inflammation on intestinal tumorigenesis in the absence of a functional mismatch repair system, IL‐10^−/−^ mice were cross‐bred with MSH2^loxP/loxP Vil‐cre^ to generate MSH2^loxP/loxP Vil‐cre^ × IL‐10^−/−^ DKO mice. Mice were bred under specific pathogen‐free (SPF) conditions. A subset of 7‐9 week old mice were either moved to conventional housing or remained under SPF conditions. At 32‐34 weeks, mice were euthanized, the intestines dissected and analyzed for tumor burden and cancer stage (Figure 1) in the small and the large bowel (representative images of intestinal Swill Rolls are shown in Figure S1).

Under conventional housing, the number of mice bearing at least one tumor in the small or large bowel was not different between DKO and MSH2^loxP/loxP Vil‐cre^ mice (Figure 1A). Also for IL‐10^−/−^ mice a similar total tumor incidence was found. Under SPF conditions total tumor incidence in DKO and MSH2^loxP/loxP Vil‐cre^ was again similar, but greatly reduced in IL‐10^−/−^ mice. Under these conditions, however, tumor incidence in MSH2^loxP/loxP Vil‐cre^ mice was solely dependent on small bowel tumors (Figures 1A and S2). In contrast, 53% of DKO mice developed large bowel tumors under SPF conditions, but none of the MSH2^loxP/loxP Vil‐cre^ mice and only one out of 12 IL‐10^−/−^ mice (Figure 1A,B). Under conventional housing tumor development in the large bowel of DKO mice was comparable to that of SPF housed mice with an increase to 78% in DKO mice compared to 8% in MSH2^loxP/loxP Vil‐cre^ mice (*P* = .002) and 40% in IL‐10^−/−^ mice (Figure 1B), indicating that intestinal tumorigenesis in DKO mice was independent of housing conditions. In contrast, tumorigenesis in IL‐10 ^−/−^ mice was dependent on housing conditions.

DKO mice displayed the highest incidence of large bowel carcinomas, both under conventional housing and SPF conditions (Figure 1C). MSH2^loxP/loxP Vil‐cre^ mice had no colonic carcinomas at all. Looking at tumor multiplicity in the large intestine, we saw an increase in tumor numbers in DKO mice compared to MSH2^loxP/loxP Vil‐cre^ mice (*P* = .003, Figure 1D) under conventional housing. Similarly, the number of colonic tumors in DKO mice increased tremendously compared to single KO mice under SPF conditions (*P* = .025 compared to MSH2^loxP/loxP Vil‐cre^ and *P* = .032 compared to IL‐10^−/−^ mice, Figure 1D). This rise in colonic tumors in DKO mice was predominantly due to the development of proximally located tumors, including the proximal third of the colon, the ileocecal valve and the cecum, rather than distal tumors, including the two distal thirds of the colon and the rectum (Figure 1E,F). In conclusion, under mismatch repair deficiency, IL‐10 deletion aggravates tumorigenesis specifically in the large intestine. Tumor development in DKO mice predominates in the proximal colon.

### 3.2 Intestinal inflammation is aggravated in DKO mice

**FIGURE 2:**
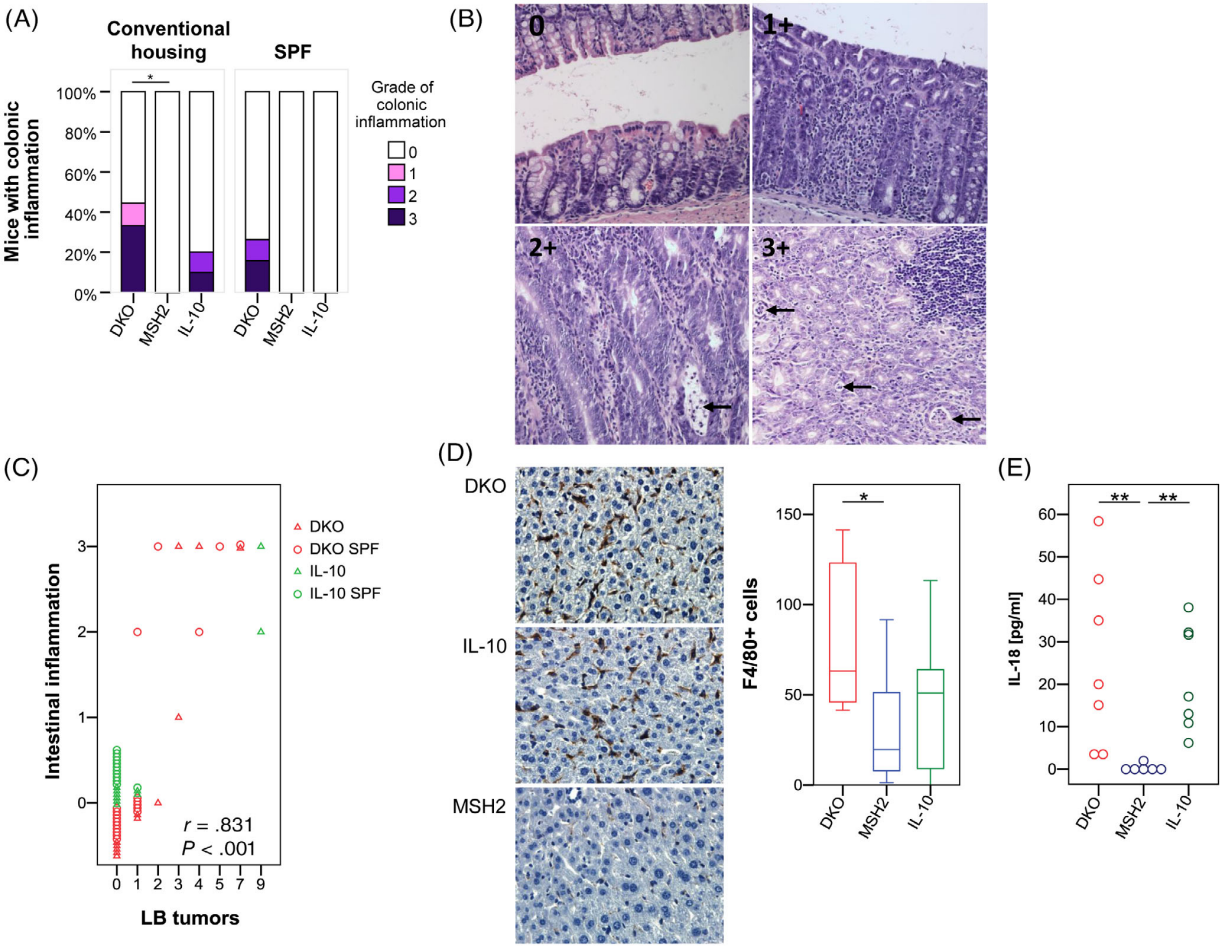
Intestinal inflammation and hepatic Kupffer cells. A, Grade of colonic inflammation was evaluated for all mice and housing conditions. B, Representative H&E stainings of (0) uninflamed mucosa, (1+) with patchy inflammatory infiltrate, moderately inflamed with low number of crypt abscesses and (3+) highly inflamed colonic epithelial tissue with high number of crypt abscesses in DKO mice. C, DKO or IL‐10^−/−^ mice with intestinal inflammation showed increased numbers of large bowel tumors (Pearson's correlation *r* = .831, *P* < .001), an effect independent of genotype and housing conditions with regard to DKO mice. D, Exemplary images of F4/80^+^ liver macrophages for all genotypes from conventionally housed mice at ×400 magnification. Liver macrophages were increased in DKO mice (n = 8) compared to MSH2^loxP/loxP Vil‐cre^ mice (n = 11), but not IL‐10^−/−^ mice (n = 8; *P* = .016 and *P* = .195, respectively). E, IL‐18 serum levels were elevated in DKO mice (n = 7; *P* = .001) and IL‐10^−/−^ mice (n = 7; *P* = .001) compared to MSH2^loxP/loxP Vil‐cre^ mice (n = 6). Statistical analysis: Pearson's χ^2^‐test for inflammation incidence; Pearson's correlation coefficient to correlate LB tumors and intestinal inflammation; Independent samples Mann‐Whitney *U* test for F4/80^+^ cells and IL‐18 serum levels; *P < .05; **P < .01

IL‐10^−/−^ mice are a model for human inflammatory bowel disease and develop inflammation in the distal ileum and proximal colon. The proximal colon is also the site where they develop most colonic tumors. In our model, we scored intestinal inflammation by evaluating the extent of immune cell infiltration and number of crypt abscesses. Four out of nine DKO mice under conventional housing developed large bowel inflammation and five out of 19 DKO mice kept under SPF conditions, whereas only two out of 10 IL‐10^−/−^ mice under conventional housing developed inflammation and none of the IL‐10^−/−^ mice kept under SPF conditions (Figure 2A,B). As expected, MSH2^loxP/loxP Vil‐cre^ mice did not develop intestinal inflammation. As previously observed, intestinal inflammation in IL‐10^−/−^ mice is dependent on housing conditions^11^; however, in DKO mice, large bowel inflammation occurs independent of housing conditions and to a higher degree as compared to IL‐10^−/−^ mice.

As chronic intestinal inflammation can lead to tumor formation, the relationship between the degree of intestinal inflammation and large bowel tumorigenesis was analyzed for IL‐10^−/−^ and DKO mice (Figure 2C). Indeed, mice with a high degree of inflammation presented with the highest tumor numbers (Pearson's correlation *r* = .831, *P* < .001). For DKO mice, this relationship is independent of housing conditions.

In conclusion, inflammation exacerbates colon carcinogenesis in the absence of a functional mismatch repair system independent of housing conditions. Tumorigenesis and inflammation are suppressed in IL‐10^−/−^ mice under SPF conditions, which is not the case for DKO mice.

### 3.3 DKO mice have an increase in F4/80^+^ hepatic Kupffer cells and serum IL‐18 levels

Translocation of intestinal bacteria and bacterial products into the liver as a result of impaired gut barrier function can lead to an increase in Kupffer cells, that is, F4/80^+^ liver macrophages.^30^ Conventionally housed DKO mice had a threefold increase of F4/80^+^ macrophages with a median number of 63 F4/80^+^ cells each ×40 FoV compared to 20 in MSH2^loxP/loxP Vil‐cre^ mouse livers (Figure 2D). IL‐10^−/−^ mice presented with a median number of 51 Kupffer cells. Analysis of lobular and portal inflammation from conventionally housed mice did not reveal difference between genotypes (Figure S3), but a correlation analysis of intestinal mucosal or fecal bacteria revealed taxa which positively or negatively correlated with lobular inflammation (Figures 4C and S5). Within mucus samples, *Alistipes* negatively correlated with lobular inflammation but *Mucispirillum* and certain *Lachnospiraceae* family members showed a positive correlation with lobular inflammation. Within stool samples, *Bacteroides* and *Parabacteroides* genera were positively correlated with lobular inflammation.

Upon inflammation and intestinal barrier disruption IL‐18 production is upregulated in epithelial cells as well as macrophages and dendritic cells of the lamina propria.^31^, ^32^ Thus, serum IL‐18 levels were investigated as a marker for epithelial barrier dysfunction and systemic inflammation. Serum IL‐18 levels were increased in DKO mice and IL‐10^−/−^ compared to MSH2^loxP/loxP Vil‐cre^ (Figure 2E). In summary, the accumulation of Kupffer cells and an increase in serum IL‐18 levels in DKO mice likely reflects a higher intestinal permeability, a higher amount of translocating bacterial products and low‐grade systemic inflammation.

### 3.4 Effect of treatment on tumor incidence and intestinal inflammation

To evaluate the anti‐inflammatory and antitumorigenic effect of established and novel drugs for the treatment of ulcerative colitis, DKO mice were either fed with 500 mg 5‐ASA/kg chow or gavaged three times a week with 30 mg tofacitinib/kg body weight for 25 weeks. H&E stainings of dissected intestines were evaluated for tumor number and invasiveness. None of the two treatment regimens altered small or large bowel tumorigenesis, carcinoma formation, intestinal inflammation or microbiota composition in DKO mice, as analyzed by 16S rRNA gene sequencing of microbial profiles from stool and mucus‐enriched samples (Figure S4).

### 3.5 Crypts of proximal tumors present with more mucus‐residing bacteria than distal tumors

**FIGURE 3:**
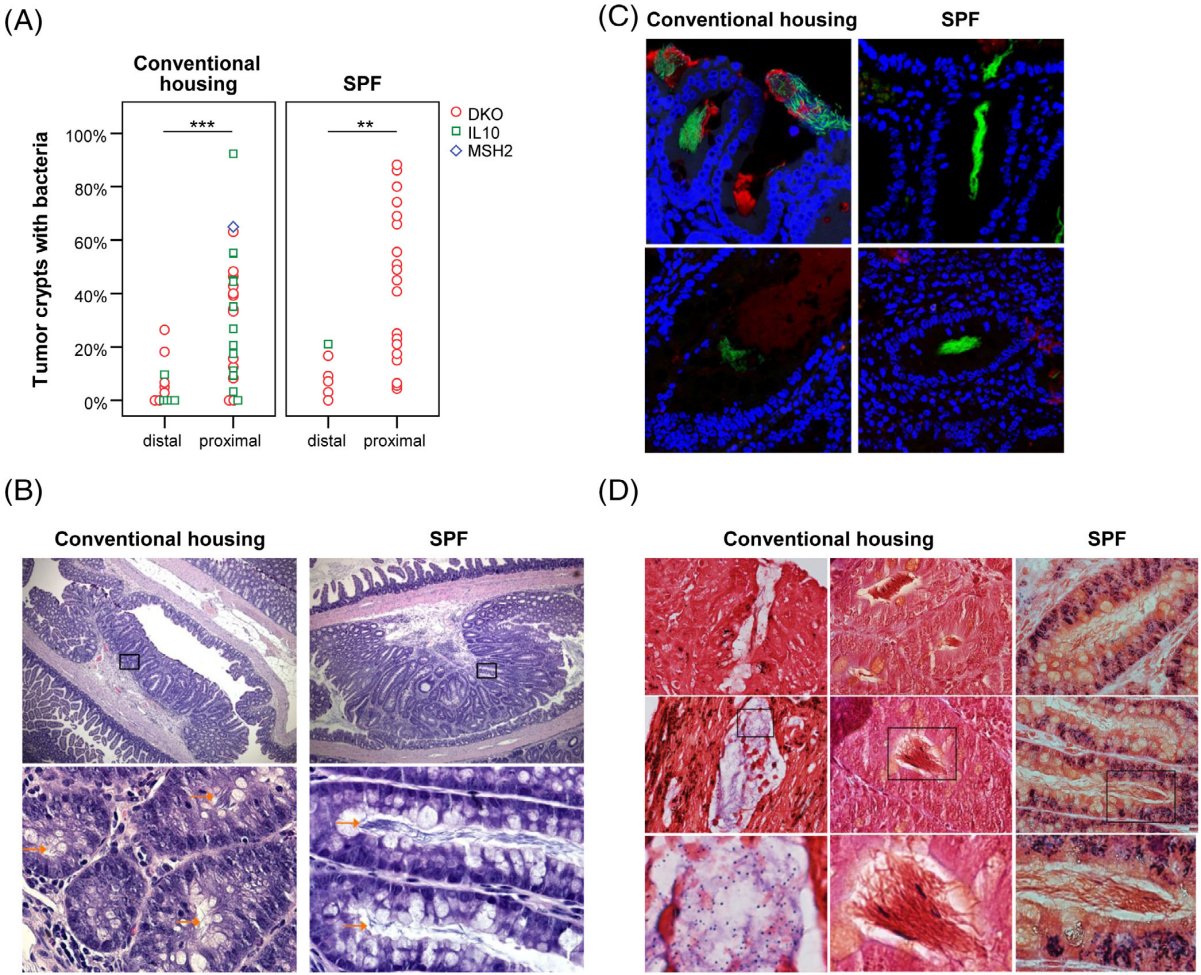
Increase of mucus‐residing bacteria in proximal tumors. A, The number of tumor crypts colonized by bacteria is higher in the proximal colon (interfold region of the large intestine, including the ileocecal valve and the cecum) compared to the distal colon (the two distal thirds of the colon including the rectum), independent of housing conditions (for DKO mice) or genotype. B, Exemplary images (top panels ×40, bottom panels ×600 magnification) of H&E stained proximal tumors from DKO mice. Orange arrows indicate bacteria within tumor crypts. C, FISH staining using the general bacterial probe mix EUB338 I‐III (green) confirmed presence of bacteria within the mucus layer (red, mucin‐2 immunofluorescence) of tumor crypt cells (blue, Hoechst counterstain). Images were scanned using a ×60 objective. D, Exemplary Gram staining (at ×600 magnification) in DKO large intestines from conventionally housed (left and middle panels) and SPF mice (right panels) revealed mostly rod‐shaped Gram‐negative bacteria within tumor crypts. Seldom, crypts also presented with Gram‐positive cocci (left panel, bottom images) or Gram‐positive rod‐shaped bacteria. Statistical analysis: Independent‐Samples Mann‐Whitney *U* test to compare two groups (distal or proximal tumor localization); **P < .01, ***P < .001

Histological analysis revealed a prominent appearance of bacteria residing in tumor crypts (Figure 3B). An analysis of tumor localization and bacterial crypt penetrance revealed a higher presence of bacteria if the tumor was located in the proximal colon (including the interfold region of the large intestine, the ileocecal valve and the cecum, Figure 3A) compared to the distal colon (the two distal thirds of the large intestine including the rectum). This increase was independent of genotype (DKO_conventional housing_: *P* = .03; IL‐10_conventional housing_: *P* = .004) and also independent of housing conditions (DKO_SPF_: *P* = .005), suggesting that not classical pathogens are located in tumor crypts, but rather opportunistic pathogens.^33^ FISH analysis using 16S rRNA‐targeted oligonucleotide probes targeting all bacteria (EUB338 I‐III, green; Table S3) verified the presence of bacteria within tumor crypts of mice both under conventional and SPF conditions (Figure 3C). Gram staining suggested that mostly long rod‐shaped and bacilli‐formed Gram‐negative bacteria are residing in the mucus of tumor crypts (Figure 3D). Some Gram‐positive cocci and bacilli were also seen. In summary, bacterial penetrance into the mucus of tumor crypts was enhanced in proximal colonic tumors from SPF and conventionally housed mice.

### 3.6 High‐grade inflammation is associated with a shift in microbiota composition

**FIGURE 4:**
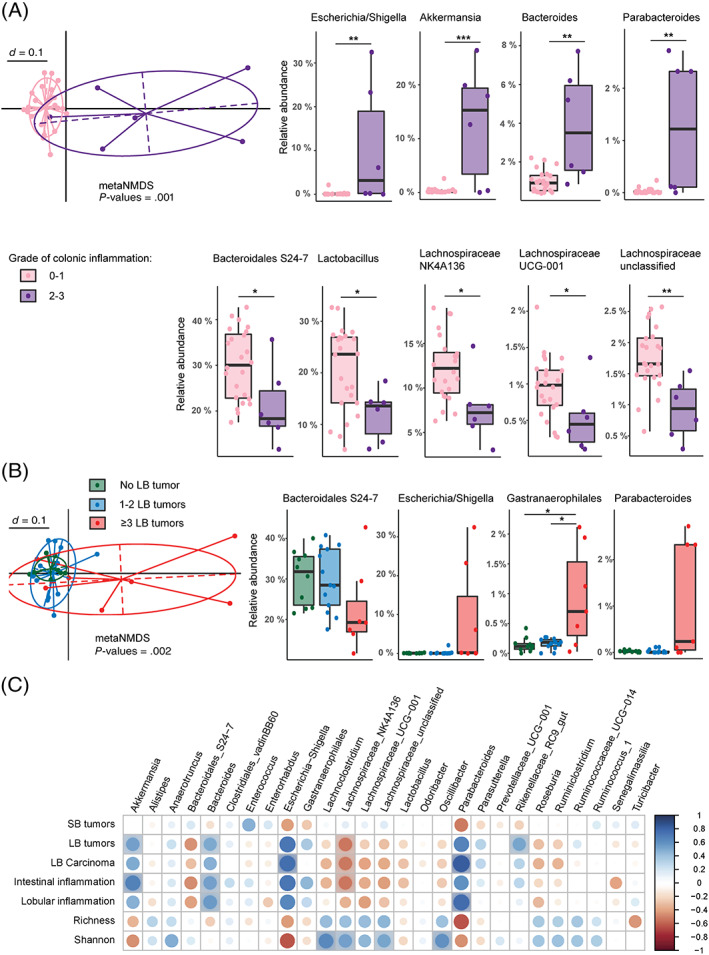
Inflammation and tumorigenesis shift fecal microbiota composition in DKO mice. A, Meta nonmetric scaling (metaNMDS) plot of microbial profiles revealed a distinct clustering of fecal microbiota composition in mice with moderate to high‐grade intestinal inflammation (grade 2 and 3) compared to mice with no or low grade intestinal inflammation (grade 0 and 1). Relative abundance plots of taxa which are significantly different between groups. B, metaNMDS plot showing a shift in stool microbiota composition of mice with a high number of large bowel tumors compared to mice with only 1‐2 or no large bowel tumors. Relative abundance plots of taxa showing trends and differences dependent on tumor count. C, Pearson's correlation analysis of bacterial genera, using stool samples from all genotypes and treatments. Blue dots indicate a positive correlation, red dots a negative correlation. Shaded circles are statistically significant correlations (corrected for multiple comparisons). Statistical analysis: pairwise Wilcoxon Rank Sum test for relative abundances, *adjusted *P*‐value ≤ .05, **adjusted *P*‐value < .01, ***adjusted *P*‐value < .001

To identify bacteria which are associated with large bowel inflammation and tumorigenesis in DKO mice, DNA was isolated from fecal samples and mucus‐enriched fractions and 16S rRNA gene amplicon sequencing from the V3‐V4 variable region was performed. For analysis, conventionally housed DKO were subgrouped into mice with moderate to high‐grade inflammation and those with weak or no inflammation. Multidimensional scaling plots of microbial profiles revealed a shift of the fecal and mucosal microbiome (Figure 4A, metaNMDS *P* = .001 and Figure S5A, *P* = .006) in mice with inflamed colons. Inflammation was associated with an increase in the relative abundances of taxa belonging to the *Escherichia‐Shigella* group, as well as *Akkermansia*, *Bacteroides* and *Parabacteroides* genus members in stool samples (Figure 4A, right panels). A significant decrease of *Bacilli* in mice with inflammation was found, resulting from the loss of the highly abundant genus *Lactobacillus* in mice with no or low‐grade inflammation. *Lachnospiraceae* and *Muribaculaceae* (*Bacteroidales S24‐7*) family members were reduced as well. Similar trends were found for *Escherichia‐Shigella* and *Lachnospiraceae NK4A136* group members in the mucus‐enriched fractions (Figure S5A). Mice with a high number of large bowel tumors (≥3) showed as well a trend for enrichment in *Escherichia‐Shigella* and *Parabacteroides* genus members and a decrease in *Muribaculaceae* family members in fecal and mucosal samples (Figures 4B and S5B). Correlation analysis of fecal bacterial taxa (from all genotypes and treatment groups of conventionally housed mice) revealed a positive correlation of large bowel tumors with *Akkermansia*, *Bacteroides* and *Rikenellaceae* family members and large bowel carcinomas with *Escherichia‐Shigella* and *Parabacteroides* (Figure 4C). Similarly, a positive correlation for intestinal inflammation and abundance of *Akkermansia* and *Bacteroides* is evident. *Lachnospiraceae NK4A136* group was the only bacterial taxa negatively associated with tumorigenesis and inflammation (Figure 4C and Figure S5C) which is also evident in abundance plots. For mucus samples, a positive correlation between *Escherichia‐Shigella* and intestinal inflammation as well as *Mucispirillum* and large bowel carcinogenesis is present. In summary, large intestinal inflammation and a high number of large bowel tumors is associated with a shift of the fecal and mucosal microbiome in DKO mice.

## 4 DISCUSSION

Here we studied the relevance of inflammation, intestinal tumor localization and bacterial proximity in a novel mouse model, crossing MSH2^loxP/loxP Vil‐cre^ mice with IL‐10^−/−^ mice. In DKO mice tumors are mainly located in the proximal colon, display a high proportion of cancer and link to the degree of inflammation, as well as presence of bacteria in tumor crypts. Thus, under chronic inflammatory conditions mismatch repair deficiency aggravates colonic tumorigenesis with invasion of mostly rod‐shaped bacteria into the mucus of tumor crypts.

It has been shown for MSH2^loxP/loxP Vil‐cre^ mice that the number of mutations per microsatellite marker, as a measure of microsatellite instability (MSI), is increased in microdissected tumor tissue compared to normal intestinal epithelial tissue in these mice.^14^ In humans, MSI‐tumors often present in the proximal colon.^17^, ^34^ Also, patients with inflammatory bowel diseases more often develop tumors in the proximal colon, a finding that lacks good explanation.^3^ The proximal colon of mice is rather loose and thus penetrable by bacteria in the initial state without any disease.^35^ From other mouse models it is known that the presence of bacteria in close proximity to the intestinal epithelium can cause large bowel inflammation. Chronic inflammation itself is a precancerous condition.^11^ Germ‐free or antibiotics‐treated IL‐10^−/−^ mice, however, neither develop enterocolitis nor tumors.^36^, ^37^ The presence of intestinal microbiota in our DKO mice is likely also the cause of inflammation and tumor development.

Specific bacterial strains in the intestinal tract can lead to hyperproliferation by WNT‐pathway activation, to DNA damage by bacterial toxins and to inflammation in the adjacent mucosa by destruction of the mucus layer enabling bacterial‐epithelial contacts. *Akkermansia*, one of the best known mucus degraders, belonging to the class of *Verrucomicrobiae* was enriched in mice with colonic inflammation and showed a positive correlation with large bowel tumorigenesis (Figure 4). This positive association between enrichment in *Akkermansia*, inflammation and tumorigenesis has also been found in other models of colitis‐associated carcinogenesis such as the AOM/DSS model,^7^ inflammation models such as the IL10^−/‐^NLRP6^−/−^ mice^32^ and infection models.^38^ For example, monocolonization with *Akkermansia muciniphila* is sufficient to drive spontaneous colitis development in germ‐free IL10^−/−^ mice.^32^ Authors conclude that *A. muciniphila* can act as pathobiont.

In humans, fecal samples from patients with a carcinoma are enriched in species belonging to the genus *Bacteroides*, *Parabacteroides* and *Escherichia*, besides others, compared to patients with adenomas and healthy controls.^39^ These bacterial taxa are also associated with inflammation and carcinogenesis in our mouse model.

Correlation analysis and relative abundance data revealed *Lachnospiraceae NK4A136* group members and *Muribaculaceae* (*Bacteroidales S24‐7*) family members as the only taxa to be inversely correlated with inflammation and carcinogenesis. *Lachnospiraceae* family members are known butyrate producers and are less abundant in feces of colorectal cancer patients compared to healthy volunteers.^40^

A shortcoming of MSH2^loxP/loxP Vil‐cre^ mice is that large bowel tumorigenesis is rather low, as these mice predominately develop small intestinal tumors.^14^ However, with our DKO mice, which include three important factors of colon carcinogenesis, that is, mismatch repair deficiency, inflammation and bacterial proximity, colonic tumorigenesis may better resemble the human condition. One limitation of the study is the fact that we cannot conclude whether presence of bacteria in the mucus of tumor crypts is a consequence or an initiating factor of tumor development. Data from other researchers points to a mutagenic mucosal biofilm microbiota which can be transmitted from humans to tumor‐susceptible mice.^4^ Which microbes are residing in the mucus of tumor crypts and whether mucus‐residing microbes from our DKO mice might have a similar carcinogenic effect still needs to be determined. Further experiments in germ‐free mice, to verify the importance of inflammation and the proximity of bacteria to the mucosal surface for colonic tumorigenesis in DKO mice, would be needed to finally prove our hypothesis. In depth analysis of tumor‐crypt bacteria and cultivation of such bacteria will give further insights into the physiological and pathogenic role in the gut.

## CONFLICT OF INTEREST

The authors disclose no conflict of interest.

## ETHICS STATEMENT

All animal experiments were approved by the Austrian Federal Ministry for Science and Research (approval number: BMWF‐66.009/0324‐WF/V/3b/2016).

## Supporting information


**Appendix S1**. Supporting Information.

## Data Availability

Sequencing data have been deposited at the National Center for Biotechnology Information (NCBI) Sequence Read Archive under the BioProject Number PRJNA610647.
