# Supplementary material for: Crypt residing bacteria and proximal colonic carcinogenesis in a mouse model of Lynch syndrome
Source: Int J Cancer. 2020 May 18;147(8):2316–26. doi: 10.1002/ijc.33028 (PMC7496850; doi:10.1002/ijc.33028)
Supplement: Supplementary file 1 — Appendix S1. Supporting Information. [file IJC-147-2316-s001.pdf]

Supplementary Material for

Crypt residing bacteria and proximal colonic carcinogenesis in a mouse model of Lynch syndrome

Michaela Lang, Maximilian Baumgartner, Aleksandra Rożalska, Adrian Frick, Alessandra Riva, Michael Jarek, David Berry and Christoph Gasche

Table of Contents

Supplementary Table 1. Primers for genotyping and 16S rRNA gene sequencing 2

Supplementary Table 2. Antibody list...3

Supplementary Table 3. FISH probes 4

Supplementary Figure 1. Intestinal Swiss Rolls...5

Supplementary Figure 2. Small bowel tumor incidence. 6

Supplementary Figure 3. Hepatic inflammation. 7

Supplementary Figure 4. Neither 5-ASA nor tofacitinib affect intestinal tumorigenesis and inflammation. 8

Supplementary Figure 5. Inflammation and tumorigenesis shift mucosal microbiota composition in DKO mice. 9

Supplementary references 11

Supplementary Table 1. Primers for genotyping and 16S rRNA gene sequencing

Primer name	Primer sequence (5'->3')	Gene	Reference
184F	TACTGATGCGGGTTGAAGG	MSH2	[1]
184R	AACCAGAGCCTCAACTAGC		
165R	GGCAAACTCCTCAAATCACG		
IL-10T1.4 SENSE primer	GCCTTCAGTATAAAAGGGGGACC	IL-10	[2]
IL-10T2.2 AS primer	GTGGGTGCAGTTATTGTCTTCCCG		
IL-10 KO “Neo” 5 AS primer	CCTGCGTGCAATCCATCTTG		
oIMR1084 (forward)	GCGGTCTGGCAGTAAAAACTATC	Cre	[3]
oIMR1085 (reverse)	GTGAAACAGCATTGCTGTCACTT		
36b4 f	CATGGTGTTCTTGCCCATCAG	36b4	[4]
36b4 r	GCTTCATTGTGGGAGCAGACA		
341F, S-D-Bact-0341- b-S-17	CCTACGGGNGGCWGCAG	16S	[5]
785R, S-D-Bact-0785-a-A-21	GACTACHVGGGTATCTAATCC		
Head sequence	GCTATGCGCGAGCTGC		

Supplementary Table 2. Antibody list

Antibody	Dilution	Application	Company	Catalog number
F4/80 (BM8)	1:1000	IHC	Affymetrix	14-4801-82
Biotinylated goat anti-rat IgG antibody	1:200	IHC (2ndary antibody)	Vector Laboratories	BA-9400
Mucin-2 (F-2)	1:100	Immunofluorescence	Santa Cruz	sc-515032
Rabbit anti-mouse IgG Superclonal, Alexa Fluor 594	1:1000	Immunofluorescence (2ndary antibody)	Thermo Fisher Scientific	A27027

Supplementary Table 3. FISH probes

Probe name	Specificity	Probe sequence (5'->3')	Fluorophore	FA (%)	Reference
EUB338-I	most Bacteria	GCTGCCTCCCGTAGGAGT	6-FAM	0-50	[6]
EUB338-II	*Planctomycetales*	GCAGCCACCCGTAGGTGT	6-FAM	0-50	[7]
EUB338-III	*Verrucomicrobiales*	GCTGCCACCCGTAGGTGT	6-FAM	0-50	[7]

Suppl. Fig. 1

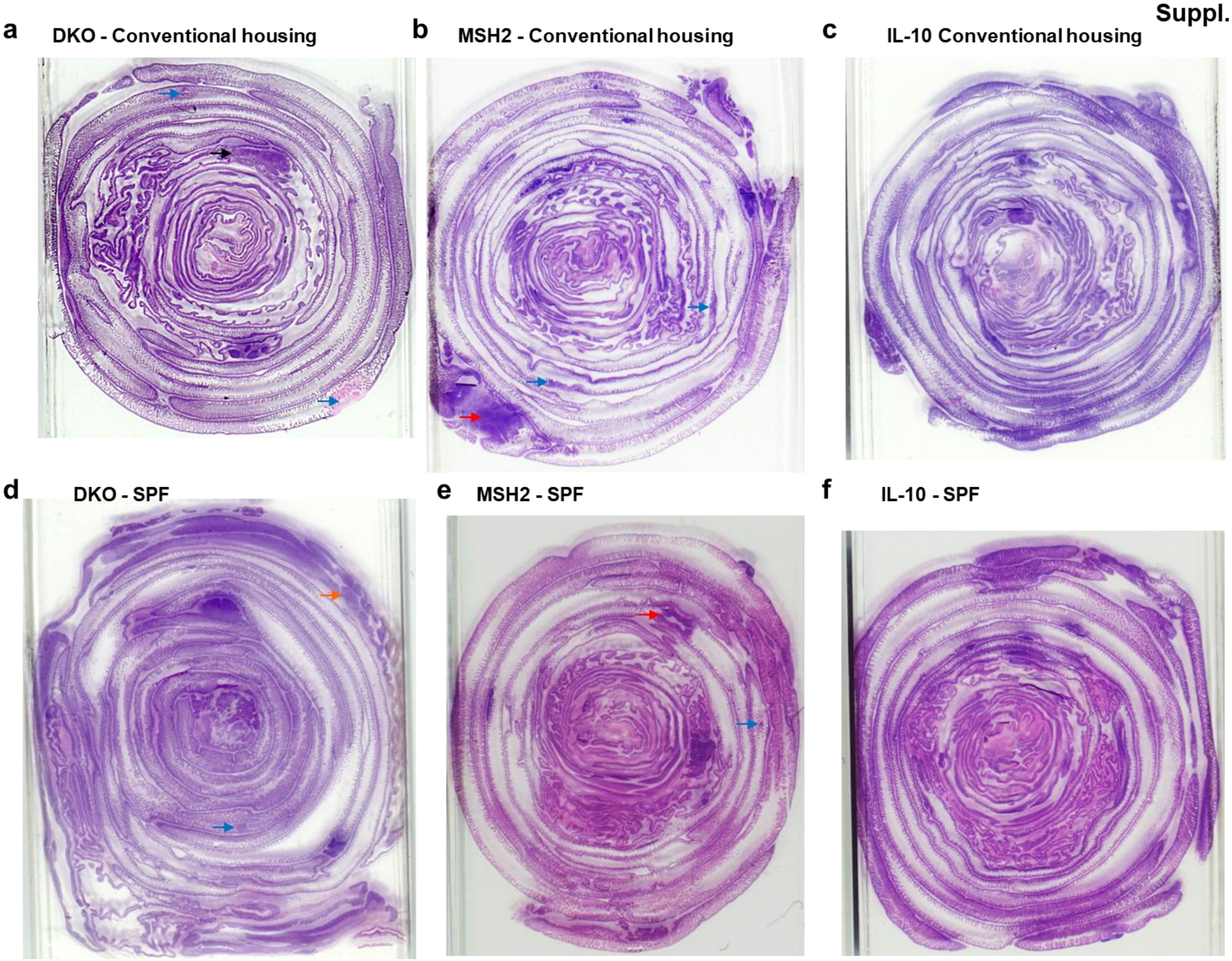

Supplementary Figure 1. Intestinal Swiss Rolls.

Representative images of H&E stained histological sections of Swiss Rolls from the small and large intestine of (a) a DKO mouse, (b) a MSH2$^{loxP/loxP\ Vill\text{-}cre}$ mouse and (c) an IL-10$^{-/-}$ mouse under conventional housing. Intestinal Swill Roll of (d) a DKO mouse, (e) a MSH2$^{loxP/loxP\ Vill\text{-}cre}$ mouse and (f) an IL-10$^{-/-}$ mouse under SPF conditions. The black arrow indicates an adenoma in the proximal large bowel, the blue arrows indicate an adenoma in the small intestine, the orange arrow indicates an invasive carcinoma in the proximal large bowel, and red arrows indicate a carcinoma in the small bowel. Swiss Roll images without an arrow do not show any tumor. Except for the DKO mouse out of SPF housing, all intestines have been coiled up with the small bowel outward and the large bowel inward.

Suppl. Fig. 2

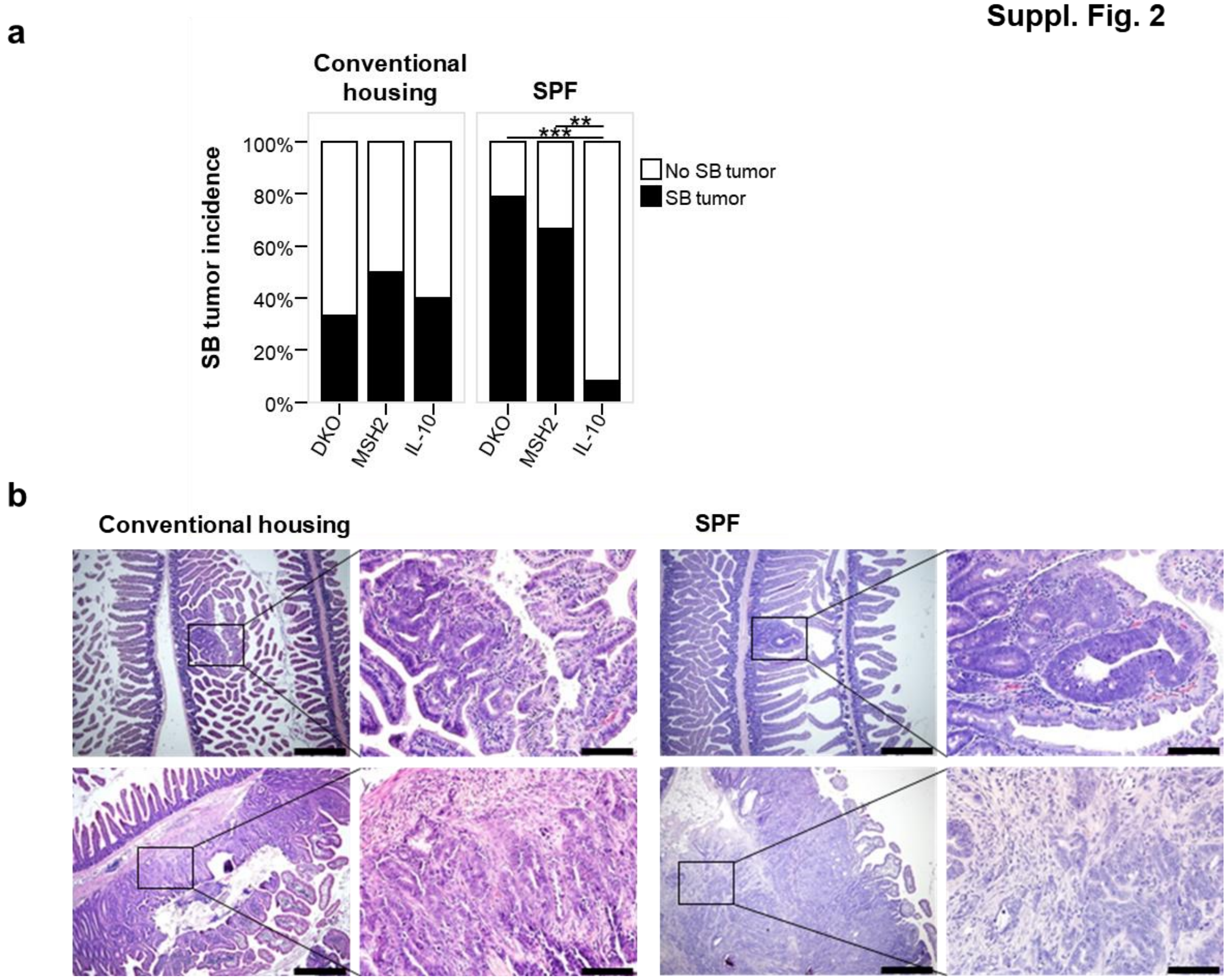

Supplementary Figure 2. Small bowel tumor incidence.

(a) Small bowel (SB) tumor incidence of conventionally housed mice was not different between genotypes. Overall, SB tumor incidence differed between genotypes under SPF conditions (p=0.001). SB tumor incidence was higher in DKO and MSH2$^{loxP/loxP\ Vil\text{-}cre}$ mice compared to IL10$^{-/-}$ mice ($p<0.001$ and $p=0.005$, respectively). (b) Representative images of adenomas (top panels) and carcinomas (bottom panels) in the small intestine from H&E stained histological sections of DKO mice from different housing conditions (Scale bars: low magnification images 500 µm; high magnification images: 100 µm). Statistical analysis: Pearson χ^2-test; **p<0.01, ***p<0.001.

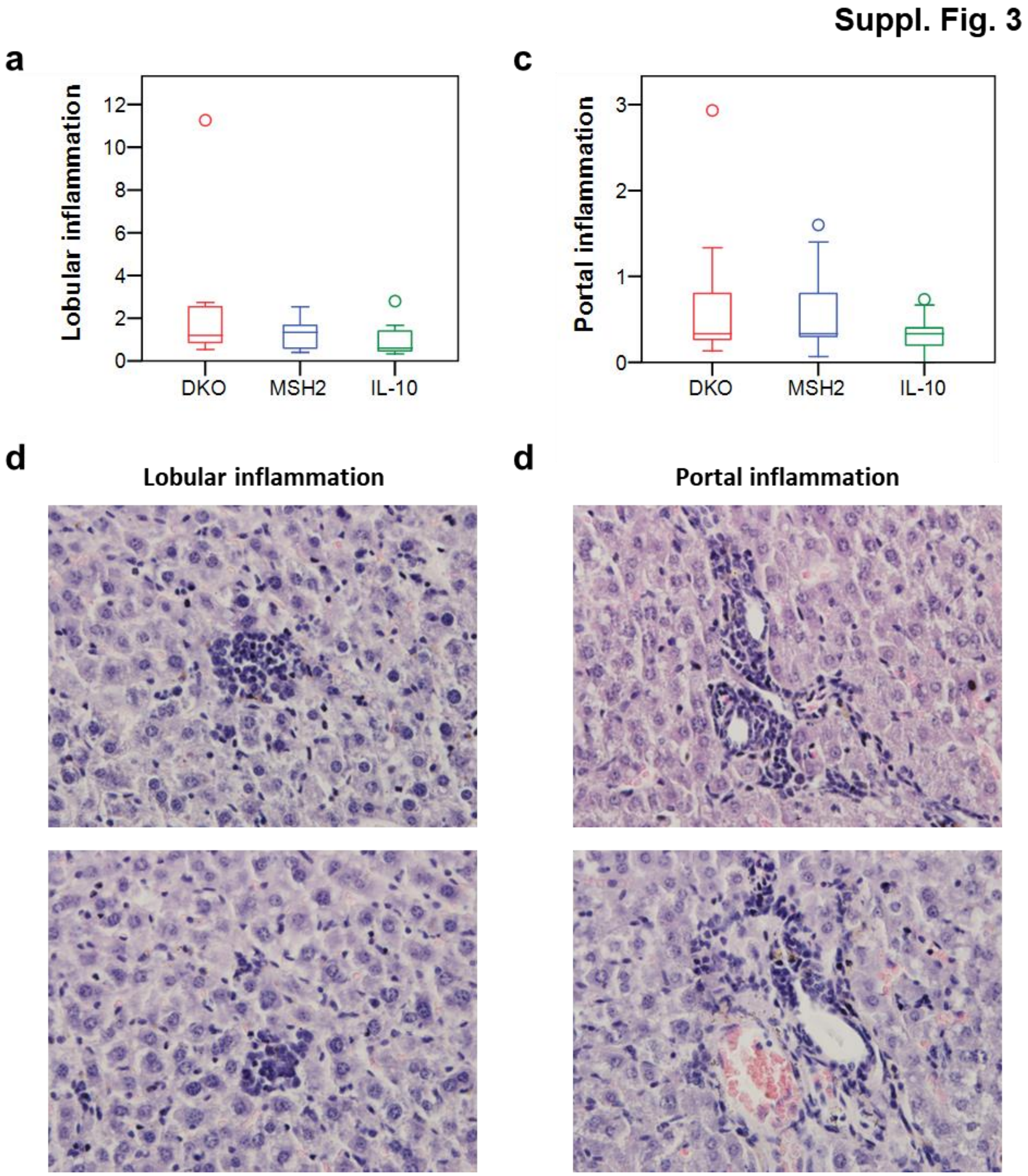

Supplementary Figure 3. Hepatic inflammation.

15 FoVs from H&E stained mouse livers (N_{DKO}=9, N_{IL10}=9, N_{MSH2}=12) were analyzed for the number of inflammatory foci with ≥ 5 inflammatory cells/focus at a magnification of 200x (a, b). For the same H&E stained livers sections, 15 portal tracts were analyzed for the presence of portal inflammation (c, d). No differences between the genotypes were found for lobular or portal inflammation. Statistical analysis was performed using Independent-Samples Kruskal-Wallis test.

Suppl. Fig. 4

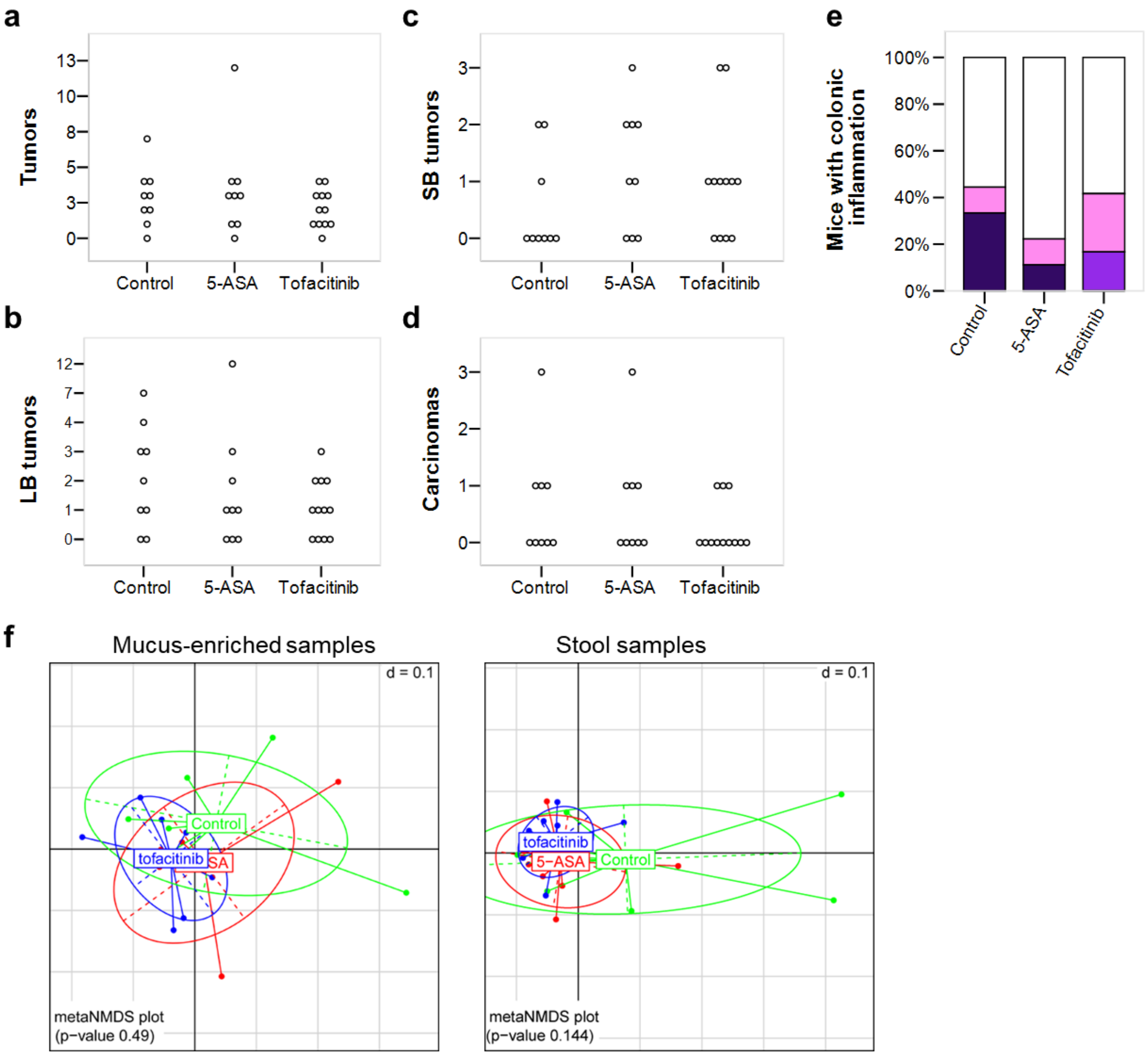

Supplementary Figure 4. Neither 5-ASA nor tofacitinib affect intestinal tumorigenesis and inflammation.

Neither 5-ASA (n=9) nor tofacitinib (n=12) altered tumor multiplicity of (a) total intestinal tumors, (b) large bowel - and (c) small bowel tumors or (d) number of carcinomas compared to sham-treated DKO mice (n=9). (e) Treatment with 5-ASA or tofacitinib did not affect intestinal inflammation. (f) Meta nonmetric scaling (metaNMDS) plots of mucus-enriched and stool samples did not show any significant differences in microbial composition upon drug treatment. Statistical analysis: Independent-Samples Mann-Whitney U test to compare tumor or carcinoma multiplicity between 2 groups; Pearson χ^2-test for inflammation incidence.

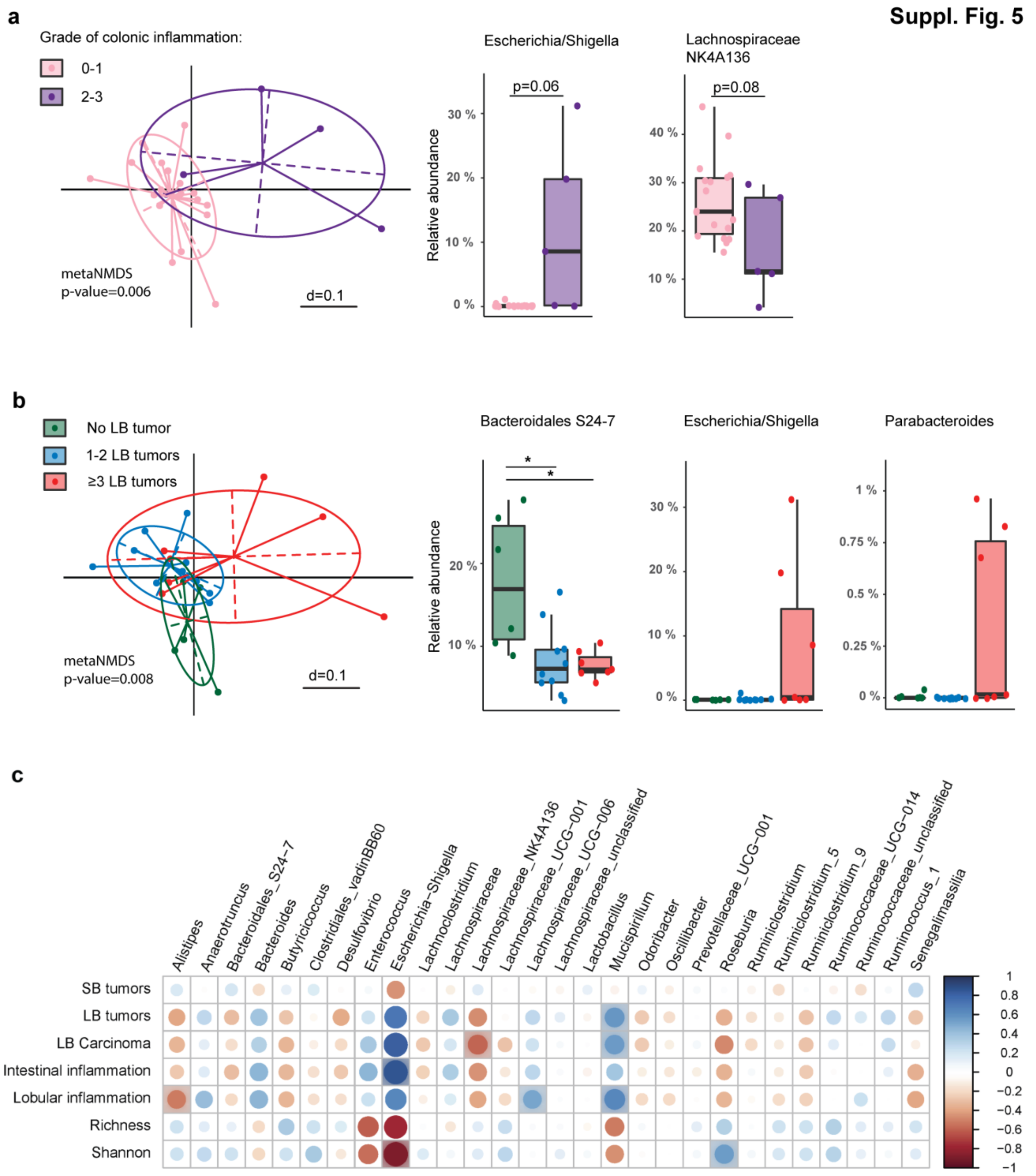

Supplementary Figure 5. Inflammation and tumorigenesis shift mucosal microbiota composition in DKO mice.

(a) Meta nonmetric scaling (metaNMDS) plot of microbial profiles revealed a distinct clustering of mucosal microbiota composition in mice with moderate to high-grade intestinal inflammation (grade 2 and 3) compared to mice with no or low grade intestinal inflammation (grade 0 and 1). Relative abundance plots of taxa which show a

trend for difference between groups. (b) metaNMDS plot showing a shift in mucosal microbiota composition of mice with a high number of large bowel tumors compared to mice with only 1-2 or no large bowel tumors. Relative abundance plots of taxa showing trends and differences dependent on tumor count. (c) Pearson's correlation analysis of bacterial genera, using mucosal samples from all genotypes and treatments. Blue dots indicate a positive correlation, red dots a negative correlation. Shaded circles are statistically significant correlations (corrected for multiple comparisons). Statistical analysis: pairwise Wilcoxon Rank Sum test, *adjusted p-value≤0.05.

Supplementary references

1. Kucherlapati MH, Lee K, Nguyen AA, Clark AB, Hou H, Jr., Rosulek A, Li H, Yang K, Fan K, Lipkin M, Bronson RT, Jelicks L, et al. An Msh2 conditional knockout mouse for studying intestinal cancer and testing anticancer agents. Gastroenterology 2010;138:993-1002 e1.
2. Kuhn R, Lohler J, Rennick D, Rajewsky K, Muller W. Interleukin-10-deficient mice develop chronic enterocolitis. Cell 1993;75:263-74.
3. Sakurai T, Kamiyoshi A, Watanabe S, Sato M, Shindo T. Rapid zygosity determination in mice by SYBR Green real-time genomic PCR of a crude DNA solution. Transgenic research 2008;17:149-55.
4. Gautherot J, Claudel T, Cuperus F, Fuchs CD, Falguieres T, Trauner M. Thyroid hormone receptor beta1 stimulates ABCB4 to increase biliary phosphatidylcholine excretion in mice. Journal of lipid research 2018;59:1610-19.
5. Klindworth A, Pruesse E, Schweer T, Peplies J, Quast C, Horn M, Glockner FO. Evaluation of general 16S ribosomal RNA gene PCR primers for classical and next-generation sequencing-based diversity studies. Nucleic acids research 2013;41:e1.
6. Amann RI, Binder BJ, Olson RJ, Chisholm SW, Devereux R, Stahl DA. Combination of 16S rRNA-targeted oligonucleotide probes with flow cytometry for analyzing mixed microbial populations. Applied and environmental microbiology 1990;56:1919-25.
7. Daims H, Bruhl A, Amann R, Schleifer KH, Wagner M. The domain-specific probe EUB338 is insufficient for the detection of all Bacteria: development and evaluation of a more comprehensive probe set. Systematic and applied microbiology 1999;22:434-44.
